# Magneto-plasmonic nanostars for image-guided and NIR-triggered drug delivery

**DOI:** 10.1038/s41598-020-66706-2

**Published:** 2020-06-22

**Authors:** Asahi Tomitaka, Hamed Arami, Arash Ahmadivand, Nezih Pala, Anthony J. McGoron, Yasushi Takemura, Marcelo Febo, Madhavan Nair

**Affiliations:** 1 Department of Immunology and Nano-Medicine, Institute of NeuroImmune Pharmacology, Centre for Personalized Nanomedicine, Herbert Wertheim College of Medicine, Florida International University, Miami, Florida 33199 USA; 2 Molecular Imaging Program at Stanford (MIPS), The James H Clark Center, Stanford University, Stanford, California 94305 USA; 3 Department of Radiology, Stanford University School of Medicine, Stanford, California 94305 USA; 4 Department of Electrical and Computer Engineering, Rice University, Houston, Texas 77005 USA; 5 Department of Electrical and Computer Engineering, Florida International University, Miami, Florida 33174 USA; 6 Department of Biomedical Engineering, Florida International University, Miami, Florida 33174 USA; 7 Department of Electrical and Computer Engineering, Yokohama National University, Yokohama, 240-8501 Japan; 8 Department of Psychiatry, McKnight Brain Institute, University of Florida College of Medicine, Gainesville, FL 32611 USA

**Keywords:** Nanobiotechnology, Nanomedicine, Nanoscale materials

## Abstract

Smart multifunctional nanoparticles with magnetic and plasmonic properties assembled on a single nanoplatform are promising for various biomedical applications. Owing to their expanding imaging and therapeutic capabilities in response to external stimuli, they have been explored for on-demand drug delivery, image-guided drug delivery, and simultaneous diagnostic and therapeutic (i.e. theranostic) applications. In this study, we engineered nanoparticles with unique morphology consisting of a superparamagnetic iron oxide core and star-shaped plasmonic shell with high-aspect-ratio gold branches. Strong magnetic and near-infrared (NIR)-responsive plasmonic properties of the engineered nanostars enabled multimodal quantitative imaging combining advantageous functions of magnetic resonance imaging (MRI), magnetic particle imaging (MPI), photoacoustic imaging (PAI), and image-guided drug delivery with a tunable drug release capacity. The model drug molecules bound to the core-shell nanostars were released upon NIR illumination due to the heat generation from the core-shell nanostars. Moreover, our simulation analysis showed that the specific design of the core-shell nanostars demonstrated a pronounced multipolar plasmon resonance, which has not been observed in previous reports. The multimodal imaging and NIR-triggered drug release capabilities of the proposed nanoplatform verify their potential for precise and controllable drug release with different applications in personalized medicine.

## Introduction

Nanomedicine, which incorporates nanomaterials for therapeutic and diagnostic applications, has been showing a rapid growth due to significant advances in nanotechnology. The progress in nanomaterials synthesis and fabrication allowed the development of nanomaterials with extraordinary multifunctionality. Among various nanomaterials developed for biomedical applications, magnetic nanoparticles are promising for a wide range of applications including drug delivery systems^1,2^, medical imaging^3,4^, bio-separation^5,6^, and biosensors^7,8^ owing to their versatile magnetic properties. Under a static magnetic field, a force is exerted on magnetic nanoparticles by a field gradient^9^. This magnetic force can be used to manipulate magnetic nanoparticles and enhance targeting efficiencies for drug delivery applications^10,11^. In contrast, magnetic nanoparticles behave differently under alternating magnetic field conditions^12^. Magnetic relaxation occurs in the presence of an alternating magnetic field and this unique response has been applied for magnetic particle imaging (MPI)^13,14^ and magnetic hyperthermia, which is a thermal treatment for cancer using heat generation from magnetic nanoparticles^15,16^. On the other hand, gold nanomaterials have demonstrated their potential for various imaging systems^17,18^, photothermal therapy^19,20^, and biosensing^21,22^ due to their tunable optical properties, great functionality, and excellent biocompatibility. Under light illumination, gold nanomaterials exhibit strong absorption of the incident light which is caused by the coherent oscillation of conduction electrons on their surface^23^. This absorption is most efficient when resonance oscillation, known as surface plasmon resonance (SPR), occurs under the electromagnetic field with the resonance frequency. This resonance strongly depends on the particle size, shape, and structure. For instance, the SPR peak red-shifts as the aspect ratio of gold nanomaterials increases^24^. The tunability of SPR is extremely important to overcome limited penetration of optical imaging systems and achieve efficient penetration into deep tissues. The near infrared (NIR) light with the wavelength ranging from 650 nm to 900 nm, referred as the first biological window, provides enhanced transparency to biological molecules and water^25,26^. Therefore, gold nanomaterials with the SPR tuned within the NIR spectra have been actively explored for advanced optical imaging systems and photothermal therapy.

Nanoparticles possessing both magnetic and plasmonic properties in a single nanosystem are highly promising for image-guided therapy. They enable more efficient treatments by incorporating imaging properties into therapeutics (*i.e*. theranostic applications). Magnetic core-gold shell nanoparticles (MNP@Au) with the SPR band in the visible region demonstrated the capacity for magnetically-guided drug delivery, as well as multimodal imaging combining magnetic resonance imaging (MRI), magnetic particle imaging (MPI) and X-ray computed tomography (CT)^27,28^. Multimodal imaging is an emerging approach which allows precise imaging by gaining synergistic effects from multiple imaging modalities^29^. It can overcome the disadvantages of each individual imaging modality by combining advantageous features from other imaging techniques.

**Figure 1:**
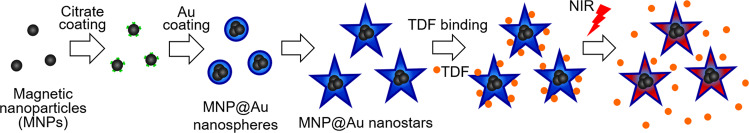
A schematic illustration of MNP@Au nanostars synthesis steps, drug binding, and NIR-triggered drug release. MNPs, magnetic nanoparticles; MNP@Au, magneto-plasmonic nanoparticles; TDF, Tenofovir disoproxil fumarate; NIR, near infrared.

Here, we propose a unique magneto-plasmonic nanostar structure consisting of a superparamagnetic iron oxide core and star-shaped plasmonic shell with high-aspect-ratio gold branches. These nanostars possess not only magnetic and plasmonic properties but also NIR-responsive property within a single nanosystem (Fig. 1). This NIR-responsive feature is advantageous for optical imaging systems and laser-assisted biomedical applications due to the high penetration efficiency of NIR light into deep tissues. We applied this feature for optical imaging system photoacoustic imaging (PAI) and NIR-triggered controlled release of therapeutic agents in addition to magnetically guided drug delivery and multimodal imaging using MRI and MPI. MRI is a medical imaging modality which acquires signals from hydrogen protons in soft tissues and provides anatomical information. MNPs are used as negative contrast agents and indirectly detected in MRI. In contrast, MPI is a pre-clinical imaging modality which acquires signals directly from the magnetization response of MNPs to an external magnetic field. MPI is capable of real-time quantitative imaging with depth-independent resolution^30^. PAI is an emerging hybrid imaging technique, which combines optical imaging and ultrasound imaging into one imaging system. It takes advantages of high optical contrast in optical imaging and high spatial resolution in ultrasound imaging, and overcomes limitations of each imaging modality^31^. By combining these advantageous properties, multimodal imaging guidance incorporating MRI, MPI, and PAI allows highly sensitive real-time tracking of therapeutic agents after administration as well as acquisition of accurate anatomical information. Moreover, NIR-triggered controlled release allows on-demand treatment at a desired location, timing, and drug release rate. These versatile properties of magneto-plasmonic nanostars are promising for personalized medicine including image-guided on-demand drug delivery. Therefore, the developed nanostars were experimentally and theoretically tested for magnetic and NIR-responsive plasmonic properties, multimodal imaging capabilities, and NIR-triggered controlled drug release.

## Results and discussion

### Synthesis and characterization of magneto-plasmonic nanostars (MNP@Au nanostars)

NIR-responsive nanostars with magnetic nanoparticle-core and gold-shell structure (MNP@Au nanostars) were synthesized using a two-step procedure. First, magnetic nanoparticles (MNPs) were synthesized by co-precipitation of Fe^2+^ and Fe^3+^ ions in an alkaline solution, followed by gold coating of MNPs by seed mediated synthesis^27,32^. MNPs and sodium citrate were used as seeds and a reducing agent for Au^3+^ ions, respectively. Core-shell nanoparticles with spherical morphology (MNP@Au nanospheres) was synthesized in this step, and the absorbance peak was observed within the visible wavelength range^27^. The morphology of the core-shell nanoparticles was modified from spherical to star shape by a reduction process using MNP@Au nanospheres as seeds, L-ascorbic acid as a reducing agent for Au^3+^ ions, and silver nitrate as an additive to induce anisotropic growth of gold. Silver ions have been employed in the seed mediated synthesis as an additive to control the shape of gold nanomaterials. It has been reported that silver ions deposit on the specific facets of Au surface and undergo reduction process via under-potential deposition^33^. The reduced silver layers on Au surface assist the anisotropic growth of Au and form branches^34^. Supplementary Information includes the absorbance spectra of MNP@Au nanostars synthesized by varying the concentrations of silver nitrate and L-ascorbic acid (Supplementary Methods, Results, and Fig. S1).

**Figure 2:**
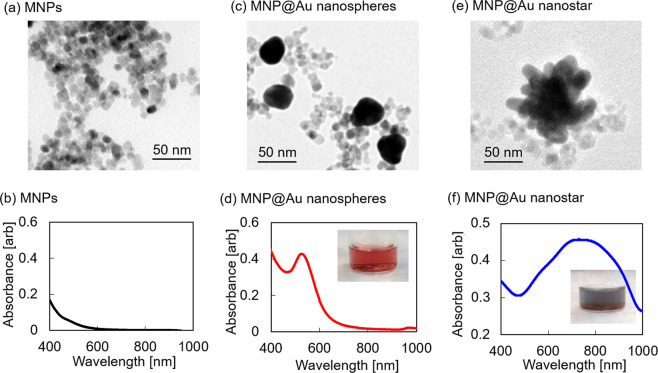
Morphology and absorbance spectra of synthesized nanoparticles. (**a**) TEM image and (**b**) absorbance spectrum of MNPs. (**c**) TEM image and (**d**) absorbance spectrum of MNP@Au nanospheres. Inset: The picture of MNP@Au nanospheres. (**e**) TEM image and (**f**) absorbance spectrum of MNP@Au nanostars. Inset: The picture of MNP@Au nanostars.

The morphology, zeta potential, and absorbance spectra of synthesized MNPs, MNP@Au nanospheres, and MNP@Au nanostars were observed using a transmission electron microscope (TEM), zeta sizer, and UV-visible spectrophotometer (UV-vis). TEM images showed the spherical shape of MNPs and MNP@Au nanospheres, and star-shaped morphology of MNP@Au nanostars (Fig. 2(a,c,e), Supplementary Fig. S2). The particle sizes of MNPs and MNP@Au nanospheres and tip-to-tip diameter of MNP@Au nanostars were 10.4 ± 2.3 nm, 33.0 ± 5.0 nm, and 89.0 ± 14.5 nm, respectively. Some uncoated MNPs with weaker contrasts and smaller sizes were also observed after gold coating. The zeta potential of MNPs and MNP@Au nanospheres and MNP@Au nanostars was measured to be −48.4 mV, −40.1 mV, and −29.3 mV, respectively. The highly negative charge of MNPs and MNP@Au nanospheres is contributed by sodium citrate on the nanoparticle surface^27^. The less negative potential was observed for MNP@Au nanostars due to the use of ascorbic acid during the morphology change step. Figure 2(b,d,f) shows absorbance spectra of MNPs, MNP@Au nanospheres, and MNP@Au nanostars. In contrast to MNPs, which did not show apparent absorbance peaks within the measurement range, MNP@Au nanospheres showed an absorbance peak within the visible wavelength range (526 nm). However, by modifying the morphology of MNP@Au from spherical to star shape, the absorbance peak of MNP@Au nanostars shifted to the NIR wavelength range (703 nm). This unique optical property of MNP@Au nanostars is based on SPR which is caused by the oscillation of conduction electrons on Au shell excited by NIR illumination^35^. Under the electromagnetic field, opposite charges accumulate along with the polarization of the incident wave, which gives rise to an electric dipole mode. The electric field induced by the electric dipole exerts a force on the electrons to move to the equilibrium position, leading to the oscillation of electrons at the resonance frequency^36^. The SPR of gold nanomaterials is highly dependent on the particle size, shape, and thickness of the shell. Spherical shape Au nanoparticles and Au shells show SPR peaks at visible wavelengths^23^. In contrast, Au nanostructures with higher aspect ratio shapes such as nanorods and nanostars red-shift the SPR peak and show the peaks within the NIR wavelength range^23^.

**Figure 3:**
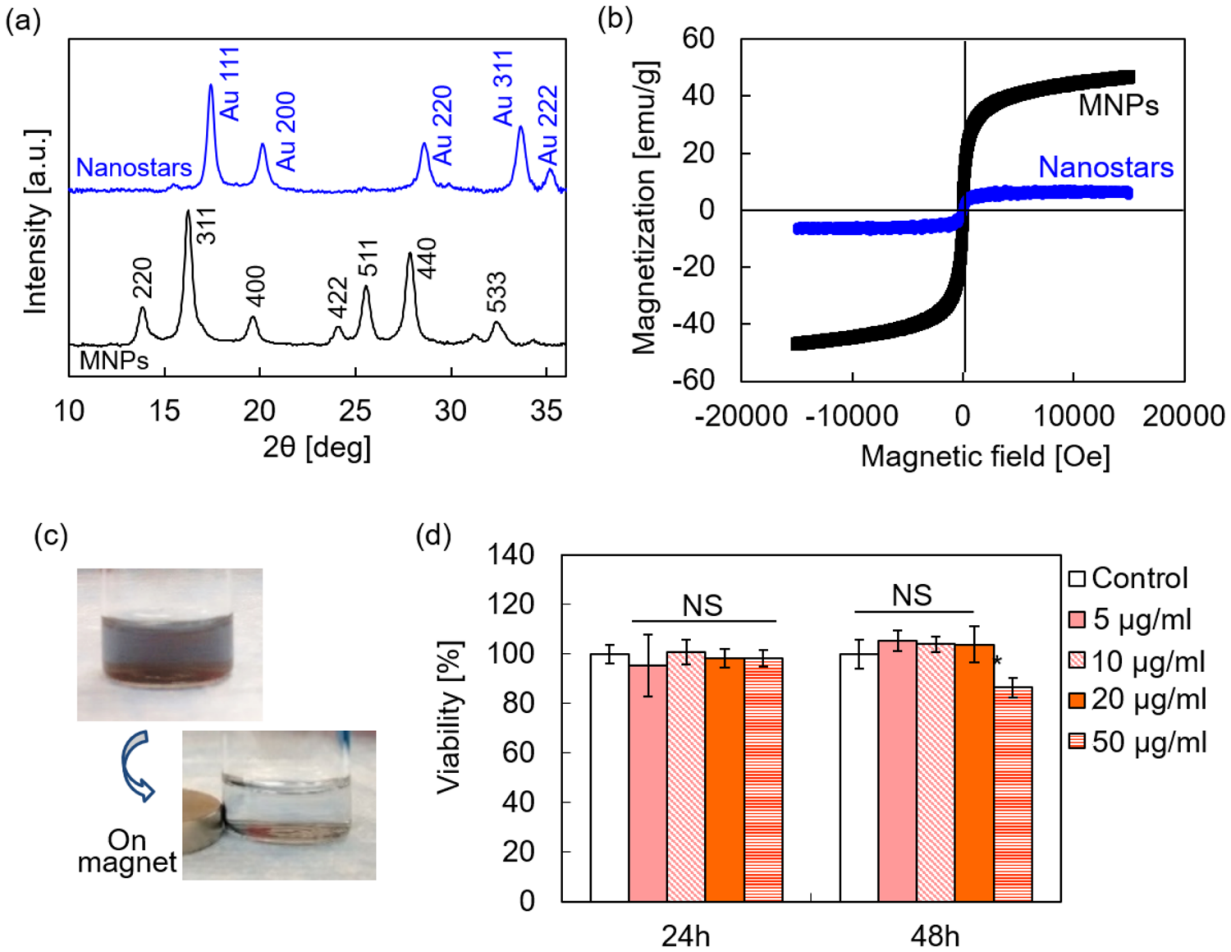
Characterization of MNP@Au nanostars. (**a**) X-ray diffraction (XRD) patterns, (**b**) magnetization curves of MNPs and MNP@Au nanostars, (**c**) the picture of MNP@Au nanostars attracted by a magnet, and (**d**) viability of CHME-5 cells after exposure to MNP@Au nanostars. (The results are represented as the mean ± standard deviation.) MNP@Au nanostars exhibited superparamagnetic property. The viability was determined using XTT assay (*P < 0.05; NS, not significant, P > 0.05).

Successful synthesis of MNP@Au nanostars was further confirmed by evaluating their crystalline structure and magnetic properties. The existence of magnetic-core and gold-shell was confirmed by analysis of X-ray diffraction (XRD) patterns as shown in Fig. 3(a) and selected area electron diffraction (SAED) patterns (Supplementary Fig. S3). The peaks from MNPs corresponded to the inverse cubic spinel phase of Fe_3_O_4_, and MNP@Au nanostars showed sharp peaks contributed from gold shell as well as smaller peaks representing Fe_3_O_4_. We previously calculated the gold shell thickness of MNP@Au nanospheres by applying Scherrer’s equation on the diffraction peaks^12^. Based on the crystallite size *d* given by

1 $$d=\frac{K\times \lambda }{\beta \times cos\theta }$$

where *λ* is the X-ray wavelength, *β* is the full-width at half-maximum of the X-ray diffraction peak, and *K* is a constant related to crystallite shape (equals to 0.90)^37,38^, the gold shell thickness was estimated to be 8.2 nm^12^. This small shell thickness compared to the particle size of MNP@Au nanospheres (33.0 nm) demonstrates that the gold shell was formed on MNPs core. Moreover, it can be estimated that a cluster of MNPs with the average size of 24.8 nm was formed within a gold shell of each MNP@Au nanosphere. The peak intensity from Au shell was predominant due to its higher atomic number and the higher thickness of Au shell compared to the size of MNPs^39,40^.

The magnetization curves of MNPs and MNP@Au nanostars and the picture of MNP@Au nanostars attracted to a permanent magnet are shown in Fig. 3(b,c). MNPs and MNP@Au nanostars showed superparamagnetic behavior, a unique property of MNPs below a certain critical size (i.e. showing no coercivity or remanent magnetization at room temperature). The saturation magnetization of MNPs and MNP@Au nanostars were 52 emu/g and 3 emu/g, respectively. An Au-to-Fe weight ratio of MNP@Au nanostars measured by ICP-MS was 10.7 (89 wt% Au, 11 wt% Fe_3_O_4_). The hydrodynamic size of MNP@Au nanostars were measured to be 90 nm with the polydispersity index (PdI) 0.301 (Supplementary Fig. S4). This hydrodynamic size fits within the size range reported to show optimal pharmacokinetic properties for *in vivo* applications^41^. These results prove successful synthesis of unique nanostars consisting of superparamagnetic iron oxide-core and plasmonic-shell and their strong NIR-responsive property.

Furthermore, we used XTT assay to evaluate the cytotoxicity of MNP@Au nanostars. Figure 3(d) shows the viability of a human microglia cell line (CHME-5) exposed to MNP@Au nanostars with concentrations ranging from 5 to 50 μg/ml for 24 h and 48 h. Incubation of the cells with nanostars for 24 h did not cause significant effect on the cell viability. The viability slightly decreased to 86% (14% reduction) compared to control after 48 h exposure at the concentration of 50 µg/ml. The reduction of cell viability by more than 30% is generally considered cytotoxic^42^. Therefore, this reduction is negligible in the matter of overall cytotoxicity.

### Multimodal imaging using MNP@Au nanostars

**Figure 4:**
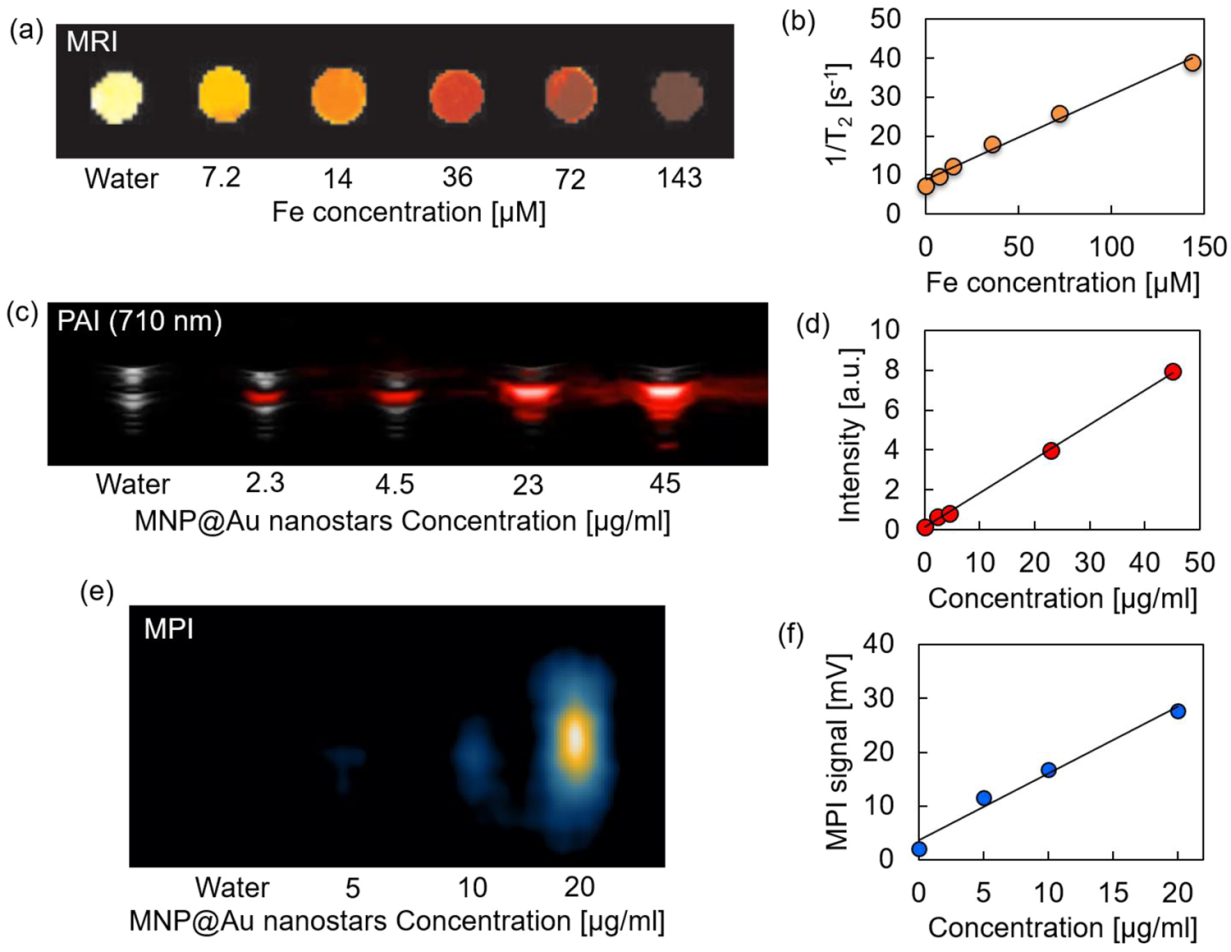
Imaging properties of MNP@Au nanostars. (**a**) T_2_-weighted MRI image, (**b**) transverse relaxivity, (**c**) PAI images, (**d**) PA intensity, (**e**) MPI images, and (**f**) MPI signals of MNP@Au nanostars. MRI images showed negative contrast with respect to nanostar concentration within the range of 2.3 µg/ml (7.2 µM-Fe) and 45 µg/ml (143 µM-Fe), and a linear trend between transverse relaxivity and Fe concentration was observed. PAI and MPI images showed concentration dependent positive contrast, and the intensity increased linearly with increasing MNP@Au nanostars concentration.

Multimodal imaging efficiency of MNP@Au nanostars was evaluated using MRI, MPI, and PAI. Figure 4(a,b) show the T_2_-weighted MR images and transverse relaxation rates of MNP@Au nanostars as a function of Fe concentration. Darker contrasts and enhanced transverse relaxation rates were observed when the nanostars concentration was increased. The transverse relaxivity was calculated to be 218 mM^−1^ s^−1^, which was higher than the transverse relaxivity of clinical T_2_ contrast agent Feridex (Ferumoxides, 133 mM^−1^ s^−1^)^43^. The relaxivity of MNPs is dependent on various parameters including particle size, composition, crystallinity, and aggregation^44^. It has been reported that transverse relaxivity of iron oxide nanoparticles was enhanced as the aggregate size increased^45^. Based on the sizes of the magnetic core (24.8 nm) of MNP@Au estimated in the previous section, the higher relaxivity could be correlated to cluster formation of magnetic cores during gold coating step.

Figure 4(c,d) show photoacoustic (PA) images and the signal intensities of MNP@Au nanostars as a function of nanoparticle concentration. MNP@Au nanostars demonstrated brighter image contrasts as the concentration increased, and the signal intensities showed a linear correlation with the nanoparticle concentration. In addition, PA images of MNP@Au nanostars at different wavelength (710 nm, 810 nm, 910 nm) are shown in Supplementary Fig. S5. The MNP@Au nanostars showed the brightest images at the wavelength of 710 nm and the contrast diminished as the wavelength increased, which corresponded with the absorbance peak of MNP@Au nanostars. Gold-based nanomaterials including gold nanorods, gold nanostars, and gold nanocages have been developed as promising contrast agents for PAI, owing to their tunable optical absorption based on SPR^46^. When the gold nanomaterials absorb optical energy, it causes rapid thermoelastic expansion and generates acoustic waves, which can be detected by ultrasonic transducers^47^. Gold nanomaterials with SPR within NIR wavelength have been extensively studied due to the minimum absorption of NIR to biological molecules^26^. Therefore, MNP@Au nanostars which exhibit the SPR peak within NIR wavelength enable efficient PAI with maximum tissue transparency.

MPI images and signal intensities of MNP@Au nanostars are also shown in Fig. 4(e,f). Brighter contrasts were observed from MNP@Au nanostars with higher concentrations. MPI signal intensities were enhanced linearly as the nanoparticle concentration increased. In contrast to MRI, MPI detects non-linear magnetization of magnetic nanoparticles resulting from dynamic magnetic responses under oscillating magnetic fields with low frequencies^48^. Since the absorption of low frequency magnetic field by human tissues is negligible, MPI provides depth-independent resolution^30^. MPI is also capable of quantitative imaging without background signal interferences due to diamagnetic properties of surrounding tissues.

### Drug binding assessment and NIR-triggered drug release

**Figure 5:**
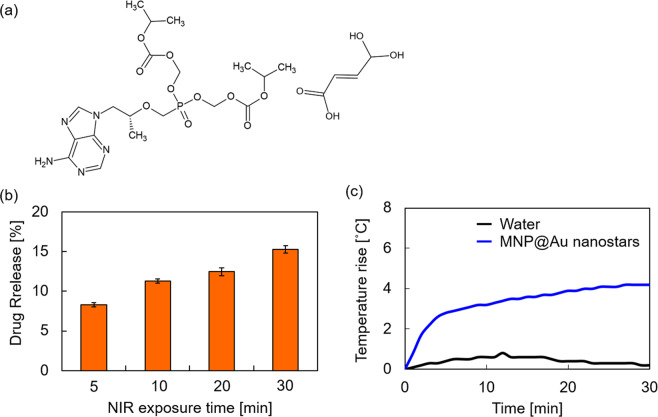
NIR induced drug release. (**a**) Chemical structure of tenofovir disoproxil fumarate (TDF). (**b**) TDF release from MNP@Au nanostars after illumination with NIR (wavelength~ 808 nm) for 5–30 min. (**c**) Temperature rise of MNP@Au nanostars solution after NIR exposure. The release of TDF from MNP@Au nanostars increased by increasing NIR exposure time. The temperature rise of MNP@Au nanostars reached 4.2 °C from baseline room temperature after NIR exposure for 30 min.

An antiretroviral drug, tenofovir disoproxil fumarate (TDF, Fig. 5(a)), was used as a model drug to demonstrate NIR-triggered drug release from MNP@Au nanostars. The capacity of TDF binding to MNP@Au nanostars was calculated to be 23 µg TDF / mg nanostars (10.4% binding efficiency) after 1 h reaction. Gold is known to have high affinity with amine groups^49,50^. It is expected that the primary amine in TDF promoted the binding of TDF to MNP@Au nanostars. The absorbance spectra of MNP@Au nanostars before and after TDF binding are shown in Supplementary Fig. S6. The absorbance peak shifted slightly after TDF binding, which indicates a negligible aggregation of MNP@Au nanostars. Figure 5(b) shows NIR-triggered release profile of TDF from MNP@Au nanostars after NIR stimulation with different exposure times. TDF release was observed as fast as 5 min of NIR stimulation and increased as the exposure time was prolonged to 30 min. Due to the SPR effect, gold nanomaterials strongly absorb incident light and convert the absorbed light into heat^23^. The oscillation of free-electrons reaches maximum at the resonance frequency of the nanomaterials, thus the heat generation of gold nanomaterials is most efficient when excited by the light with a frequency close to the resonance frequency^51^. The SPR effect caused the temperature rise of MNP@Au nanostars solution under NIR exposure at the wavelength of 808 nm. The temperature rise of the nanoparticle solution reached +4.2 °C from baseline room temperature after NIR exposure for 30 min (Fig. 5(c)). This light-to-heat conversion has been applied to NIR-triggered controlled release of drugs from gold nanomaterials^52,53^. A synergistic anticancer effect from heat-triggered release of an anticancer drug and tumor ablation has been reported when drug-loaded gold nanorods were used for cancer therapy^52^. In contrast, the heat transfer from gold nanomaterials to surrounding environment is a great concern for heat sensitive organs including the brain. By using a pulsed laser such as femtosecond laser, drug release resulting from the cleavage of the Au–S bond between gold nanomaterials and nucleic acids was achieved without heating the surrounding environment^53^. The light-to-heat conversion property of MNP@Au nanostars is a promising characteristic that can be used for NIR-triggered controlled release of different types of drugs by cleaving the bonding between the nanostars and the drug at the targeted location in body. We have also demonstrated the antiviral effect of TDF released from nanoformulation on human immunodeficiency virus (HIV)-infected microglia cells in our previous study^28^. Taken together, MNP@Au nanostars are promising nano-carriers / probes for image-guided on-demand drug delivery including HIV theranostics. As disease specificity is essential for image-guided drug delivery and theranostic applications, the future direction of this study would be to further promote cell specific targeting of MNP@Au nanostars and assess imaging capacity in cellular level.

### Calculated plasmonic response of MNP@Au nanostar

**Figure 6:**
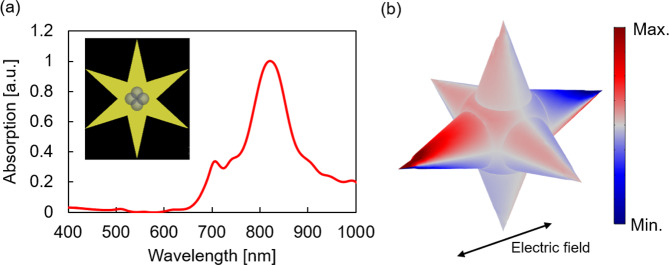
Simulation study of MNP@Au nanostars. (**a**) Normalized absorption spectra of MNP@Au nanostar under NIR illumination along with the longitudinal axis of the nanostar branches. (Inset: Geometry of MNP@Au nanostar) (**b**) Simulation results showing charge distribution inside a MNP@Au nanostar at the resonance wavelength.

The absorption spectra of a single MNP@Au nanostar were calculated using finite-difference time-domain (FDTD) and finite element method (FEM). Figure 6(a) shows the normalized absorption spectra of a single MNP@Au nanostar under NIR illumination along with the longitudinal axis of the nanostar branches based on the geometry of MNP@Au nanostar (Fig. 6, inset). A pronounced sharp absorption peak was observed around 815 nm. We observed a shorter peak wavelength and broader spectrum from experimental data compared to the calculated plasmon resonance of MNP@Au nanostar. This is likely due to the random angles between the incident light and the longitudinal axis of the nanostars in the experimental setting. Figure 6(b) depicts the corresponding charge distribution at the resonance wavelength. The longitudinal illumination against the nanostar branches excited a higher-order multipolar mode inside an MNP@Au nanostar. The dipolar moment was strongly induced along the polarization direction of the incident light, while opposite charges were formed within each tip. This charge separation along the branches and strong electric field enhancements at nanostar tips correspond to previous theoretical studies reported for gold nanostars without magnetic core^54,55^. The excitation of multipolar modes has been reported in larger and anisotropic metal nanoparticles including nanorods and nanopyramids. When the size of the nanoparticles is small enough, the electromagnetic field within the nanoparticles is uniform. However, as the particle size increases, the phase retardation of the electromagnetic field occurs within the particle, thus leading to the multipolar plasmon resonance^56,57^. In addition, the complexity of nanostar structure contributes to the excitation of multipolar modes^58^. This simulation results demonstrate a unique multipolar plasmon resonance of MNP@Au nanostars which has not been observed in previous reports. The effective optical property of MNP@Au nanostars supports the potential applicability of MNP@Au nanostars for NIR-triggered controlled drug release system.

## Materials and methods

### Synthesis method of MNP@Au nanostars

Magnetic nanoparticles (MNPs) were synthesized by co-precipitation method reported previously^32^. Briefly, an ammonium hydroxide solution was added dropwise to an aqueous solution containing iron(III) chloride hexahydrate (FeCl_3_·6H_2_O) and iron(II) chloride tetrahydrate (FeCl_2_·4H_2_O) with a molar ratio of 2:1 at 70 °C. After the reaction under stirring at 200 rpm for 30 min, a sodium citrate aqueous solution was added, and the temperature of the solution was increased to 90 °C. This reaction was continued for 30 min under stirring at 400 rpm and the synthesized citrate coated MNPs were purified with distilled water. The citrate coated MNPs were dispersed in 5 mM sodium citrate and the solution was heated to boiling temperature. After adding 85 µmol of HAuCl_4_, the reaction was continued for 6 min under stirring at 300 rpm. The resulting red-color nanoparticles (MNP@Au) were collected using a magnet and re-dispersed in distilled water.

MNP@Au nanostars were synthesized by modifying previously reported method^59^. MNP@Au (720 µg) and gold(III) chloride trihydrate (HAuCl_4_ ∙ 3H_2_O,10 µmol) were mixed under stirring at 400 rpm. An aqueous solution of silver nitrate (AgNO_3_, 3 mM) was added to the mixture followed by a dropwise addition of L-ascorbic acid solution (100 mM). The solution was stirred for 1 min and 1 M NaOH solution was added dropwise to neutralize the solution. The resulting blue-color nanoparticles (MNP@Au nanostars) were collected using a magnet and re-dispersed into distilled water and passed through a 0.2 µm filter to remove aggregates.

### Characterization of MNP@Au nanostars

Transmission electron microscope (Phillips CM-200 200 kV TEM) was operated at 80 kV to acquire the images of the nanoparticles. The absorbance spectra of MNPs and MNP@Au nanostars within the wavelength range of 400 and 1000 nm were recorded using a UV-visible spectrophotometer (Hitachi U-2910). Structural characterization and magnetization measurement of MNPs and MNP@Au nanostars were carried out using a Bruker GADD/D8 X-Ray system with a Mo anode (wavelength: 0.708 A) and a vibrating sample magnetometer (VSM-3, Toei Kogyo) at room temperature, respectively. An inductively couple plasma mass spectrometer (ICP-MS, Perkin Elmer Sciex, model ELAN DRC-II) was used to measure the iron and gold ratio of MNP@Au nanostars, which were dissolved in aqua regia prior to the measurement. The hydrodynamic size distribution and zeta potential of nanostar suspension were analyzed using a Zetasizer Nano-ZS (Malvern Instruments).

The XTT assay was conducted on CHME-5 to evaluate cytotoxicity of MNP@Au nanostars. Cells were seeded in 96-well cell culture plates (1 × 10^5^ cells per well) and incubated at 37 °C for 24 h. The cell culture medium was then replaced with fresh medium containing MNP@Au nanostars (concentrations in the range from 5 to 50 μg/ml). The cells were incubated for 24 and 48 h, and XTT/ phenazine methosulfate (PMS) mixture solution was added to each well after washing off the nanostars. After 2 h incubation, and the absorbance at 450 nm was recorded using a microplate reader (Synergy HT, multi-mode microplate reader, BioTek). Experiments were performed in quadruplicate. The data was analyzed using one-way analysis of variance (ANOVA) followed by Tukey’s multiple comparison test.

### Multimodal imaging studies

MRI, PAI, and MPI properties of MNP@Au nanostars were evaluated using a Magnex Scientific 4.7 Tesla MR scanner, a Vevo LAZR-X Imaging System (FUJIFILM VisualSonics, Inc.), and a projection Field Free Line (FFL) Momentum MPI scanner (Magnetic Insight Co.), respectively. The nanostars were fixed with 1 wt% agar and placed in polymerization chain reaction (PCR) tubes for MRI and MPI studies.

The transverse relaxation time T_2_-weighted images of MNP@Au nanostars (Fe concentrations ranging from 7.2 to 143 µM) were obtained with multi-echo multi-slice (mems) sequence using the following parameters: echo time (TE) = 10–200 at 10 ms intervals (20 TE’s total), repetition time (TR) = 3000 ms, field of view 24 mm^2^ along the read, phase directions and 1 mm along the slice direction, and data matrix of 128 × 128 × 10 slices. Signal averaging was used to increase the signal to noise. Images were imported into NIH Image J (rsbweb.nih.gov/ij) and the QuickVol plugin (http://www.quickvol.com) for processing of T_2_ maps. T_2_ maps were reconstructed from a non-linear regression of the exponential decay signal using the multi-TE value datasets. Regions of interest (ROI’s) were manually delineated using ITK SNAP program.

PA images were acquired after injecting aqueous solutions of MNP@Au nanostars in phantoms consisting of acrylic dishes with PU tubing and intralipid. The experiment was carried out using an integrated pulsed (7–10 ns) tunable optical parametric oscillator (OPO)-based laser system (a laser energy density of 20 mJ/cm^2^) and MX250 high frequency 256 element linear array transducer (21 MHz center frequency, FUJIFILM VisualSonics, Inc.). The imaging data analysis was performed using Vevo LAB software (FUJIFILM VisualSonics, Inc.).

The MPI images were acquired under the following condition: a magnetic field gradient (6, 6, and 6 T/m along x-, y- and z-axes) with an excitation field (a peak amplitude along z-axis: 20 mT at 45 kHz), overlap fraction of 90%, harmonic bandwidth at 1000 kHz, field of view (FOV) of 6 cm × 8 cm, and acquisition time of 10 seconds per projection.

### Drug binding and NIR-triggered drug release study

An antiretroviral drug, TDF was added to MNP@Au nanostars in 10 mM HEPES buffer with a weight ratio of 1:4.5. The solution was mixed for 1 h and centrifuged at 3000 g for 10 min to remove unbound drugs. The concentration of the unbound drug in the supernatant was measured using a UV-vis. The amount of drug bound to MNP@Au nanostars was calculated as follows; *W*_*Bound*_ = *W*_*Total*_ - *W*_*Supernatant*_ where *W*_*Bound*_ is the drug bound to MNP@Au nanostars, *W*_*Total*_ is the total amount of drug added during binding process, and *W*_*Supernatant*_ is the amount of drug detected in the supernatant (unbound drug).

NIR-triggered release of TDF from MNP@Au nanostars was evaluated by applying an external NIR light using a continuous wave laser (808 nm, 950 mW, Roithner Lasertechnik GmbH). The TDF bound MNP@Au nanostars was exposed to the NIR light for 5–30 min. The quantification of released TDF was conducted by separating released TDF and MNP@Au nanostars, and measuring the amount of released TDF using a UV-vis. The temperature rise of MNP@Au nanostars solution under NIR illumination was measured by an optical fiber thermometer.

### Simulation of MNP@Au nanostars

A set of 3D full-wave electromagnetic simulations were conducted both using FDTD (Lumerical 2018b) and FEM (COMSOL Multiphysics 5.3a) software tools. In all simulations, the size of the grids in all axes was set to 1 nm to precisely obtain the spectral response of MNP@Au nanostar, and perfectly matched layers (PMLs) were employed to preserve the workplace from possible destructive interferences due to the scattered beams. Particularly, in the 3D FDTD simulations, the Courant stability factor (~0.99) for the considered model was achieved by setting the simulation time steps to dt = 0.02 fs. The absorption spectra of the MNP@Au nanostar were extracted using a regular plane-wave with a pulse length of 75 fs. The complex refractive indices for Au were adopted from Johnson and Christy^60^, and the optical constants for the MNPs were attained from experimental values^35^.

For the 3D FEM simulations, to extract the charge distribution map, a frequency domain module was utilized through implementing Gauss’s theorem. The dielectric permittivity data for Au were taken from Johnson and Christy^34^, and similar to the FDTD simulations, the optical constants for the MNPs were obtained from Querry experiments^61^.

## Conclusion

NIR-responsive nanostars with magnetic core and gold-shell structure were synthesized for drug delivery applications. The MNP@Au nanostars exhibited multifunctionality combining superparamagnetic and plasmonic properties, as well as drug binding and controlled release capabilities. These nanostars generated negative contrast in T_2_-weighted MRI and positive contrasts in MPI and PAI, indicating their capacity as multimodal imaging contrast agents. The release of model drug TDF from MNP@Au nanostars was triggered upon NIR illumination due to the bonding cleavage occurring as the result of photothermal effect of the nanostars. The TDF release rate increased when the exposure time was extended to 30 min. The simulation study demonstrated a unique multipolar surface plasmon mode of the MNP@Au nanostars. Our results show that MNP@Au nanostars have promising characteristics that makes them effective candidates for image-guided drug delivery application with precise multimodal imaging capability and tunable NIR-triggered on-demand drug release.

### Supplemental Information

The following files are available as supplemental information. The materials used for this study, the method and results for synthesis optimization of MNP@Au nanostars, a TEM image of MNP@Au nanostars with higher magnification, selected area electron diffraction (SAED) patterns of MNPs and MNP@Au nanostars, and hydrodynamic size distribution of MNP@Au nanostars. (PDF)

## Supplementary information

Supplementary information.

## Data Availability

The data generated or analyzed during this study are included within the article and are available from the corresponding author upon request.
