# Supplementary material for: Magneto-plasmonic nanostars for image-guided and NIR-triggered drug delivery
Source: Sci Rep. 2020 Jun 22;10:10115. doi: 10.1038/s41598-020-66706-2 (PMC7308341; doi:10.1038/s41598-020-66706-2)
Supplement: Supplementary file 1 — Supplementary information. [file 41598_2020_66706_MOESM1_ESM.docx]

Supplementary Informaion

Magneto-plasmonic nanostars for image-guided

and NIR-triggered drug delivery

Asahi Tomitaka^1^, Hamed Arami^2,3^, Arash Ahmadivand^4^, Nezih Pala^5^, Anthony J McGoron^6^, Yasushi Takemura^7^, Marcelo Febo^8^, Madhavan Nair^1^

^1^Department of Immunology and Nano-Medicine, Institute of NeuroImmune Pharmacology, Centre for Personalized Nanomedicine, Herbert Wertheim College of Medicine, Florida International University, Miami, Florida 33199, USA

^2^Molecular Imaging Program at Stanford (MIPS), The James H Clark Center, Stanford University, Stanford, California 94305, USA

^3^Department of Radiology, Stanford University School of Medicine, Stanford, California 94305, USA

^4^Department of Electrical and Computer Engineering, Rice University, Houston, Texas 77005, USA

^5^Department of Electrical and Computer Engineering, Florida International University, Miami, Florida 33174, USA

^6^Department of Biomedical Engineering, Florida International University, Miami, Florida 33174, USA

^7^Department of Electrical and Computer Engineering, Yokohama National University, Yokohama 240-8501, Japan

^8^Department of Psychiatry, McKnight Brain Institute, University of Florida College of Medicine, Gainesville, FL 32611, USA

Correspondence and requests for materials should be addressed to M.N. (email: nairm@fiu.edu)

**Supplementary Methods**

**Materials**

Iron(III) chloride hexahydrate (ACS reagent, 97%), iron(II) chloride tetrahydrate (ReagentPlus^®^), 98%, hydrochloric acid (36.5-38.0%, BioReagent, for molecular biology), ammonium hydroxide (28% NH_3_ in H_2_O, ≥99.99% trace metals basis), sodium citrate dihydrate (≥99%, FG), gold(III) chloride trihydrate (ACS reagent, ≥49.0% Au basis), gold(III) chloride trihydrate (ACS reagent, ≥49.0%), silver nitrate, L-ascorbic acid (BioXtra, ≥99.0%), XTT sodium salt (2,3-Bis(2-methoxy-4-nitro-5-sulfophenyl)-2H-tetrazolium-5-carboxanilide inner salt), phenazine methosulfate, and intralipid were purchased from Sigma-Aldrich. Ultra pure agarose was purchased from Invitrogen. Tenofovir disoproxil fumarate (TDF) was purchased from United States Pharmacopeia. The human microglia cell line (CHME-5) was purchased from Applied Biological Materials.

**Optimization of MNP@Au nanostars synthesis condition**

MNP@Au (720 µg) and gold(III) chloride trihydrate (HAuCl_4_∙3H_2_O,10 µmol) were mixed under stirring at 400 rpm. An aqueous solution of silver nitrate (AgNO_3_) was added to the mixture followed by a dropwise addition of L-ascorbic acid solution. The solution was stirred for 1 min and 1M NaOH solution was added dropwise to neutralize the solution. The resulting nanoparticles (MNP@Au nanostars) were collected using a magnet and re-dispersed into distilled water, then passed through a 0.2 µm filter to remove aggregates. To optimize synthesis condition, the reaction was carried out with various concentrations of AgNO_3_ and L-ascorbic acid. AgNO_3_ dependence on the synthesis was evaluated by varying AgNO_3_ concentrations from 0 to 30 mM with L-ascorbic acid concentration fixed at 100 mM. The concentrations of L-ascorbic acid were also varied from 0 to 1000 mM with AgNO_3_ concentration fixed at 3 mM. The absorbance spectra of the nanoparticles were measured using a UV-visible spectrophotometer (Hitachi U-2910).

**Supplementary Results**

The synthesis condition of MNP@Au nanostars was optimized by varying the concentrations of silver nitrate and L-ascorbic acid. The pictures and absorbance spectra of MNP@Au nanostars synthesized with different conditions are shown in Supplementary Figure S1. The color of MNP@Au nanostars changed from purple to blue when the concentration of silver nitrate was increased from 0 to 3 mM. The color turned purple when silver nitrate concentration was further increased to 30 mM. The absorbance peaks of MNP@Au nanostars synthesized with the silver nitrate concentrations at 0, 0.3, 3, and 30 mM were 553 nm, 581 nm, 703 nm, and 536 nm, which corresponded with the color of MNP@Au nanostars. The variation in color and absorbance spectra were also observed for MNP@Au nanostars synthesized with different concentrations of L-ascorbic acid and the silver nitrate concentration fixed at 3 mM. The color changed from brown to blue when the concentration of L-ascorbic acid was increased from 0 to 1000 mM. The absorbance peaks of MNP@Au nanostars synthesized with the L-ascorbic acid concentrations at 0, 1, 10, 100, and 1000 mM were 567 nm, 554 nm, 886 nm, 703 nm, and 844 nm, respectively. Although the nanostars synthesized with 10 mM and 100 mM L-ascorbic acid showed high absorbance peaks within NIR wavelength region, we conducted further characterization on the nanostars synthesized with 100 mM L-ascorbic acid due to the smaller hydrodynamic size of 90 nm with the polydispersity index (PdI) 0.301. Further characterization on the MNP@Au nanostars synthesized with 3 mM silver nitrate and 100 mM L-ascorbic acid.

**Supplementary Figures**

**Figure S1.** Optimization of synthesis condition. (a) Pictures of MNP@Au nanostars synthesized with AgNO_3_ concentrations varying from 0 to 30 mM with L-ascorbic acid concentration fixed at 100 mM. (b) Pictures of MNP@Au nanostars synthesized with L-ascorbic acid concentrations varying from 0 to 1000 mM with AgNO_3_ concentration fixed at 3 mM. Absorbance spectra of MNP@Au nanoparticles synthesized with (c) varying AgNO_3_ concentrations and (d) varying L-ascorbic acid concentrations.


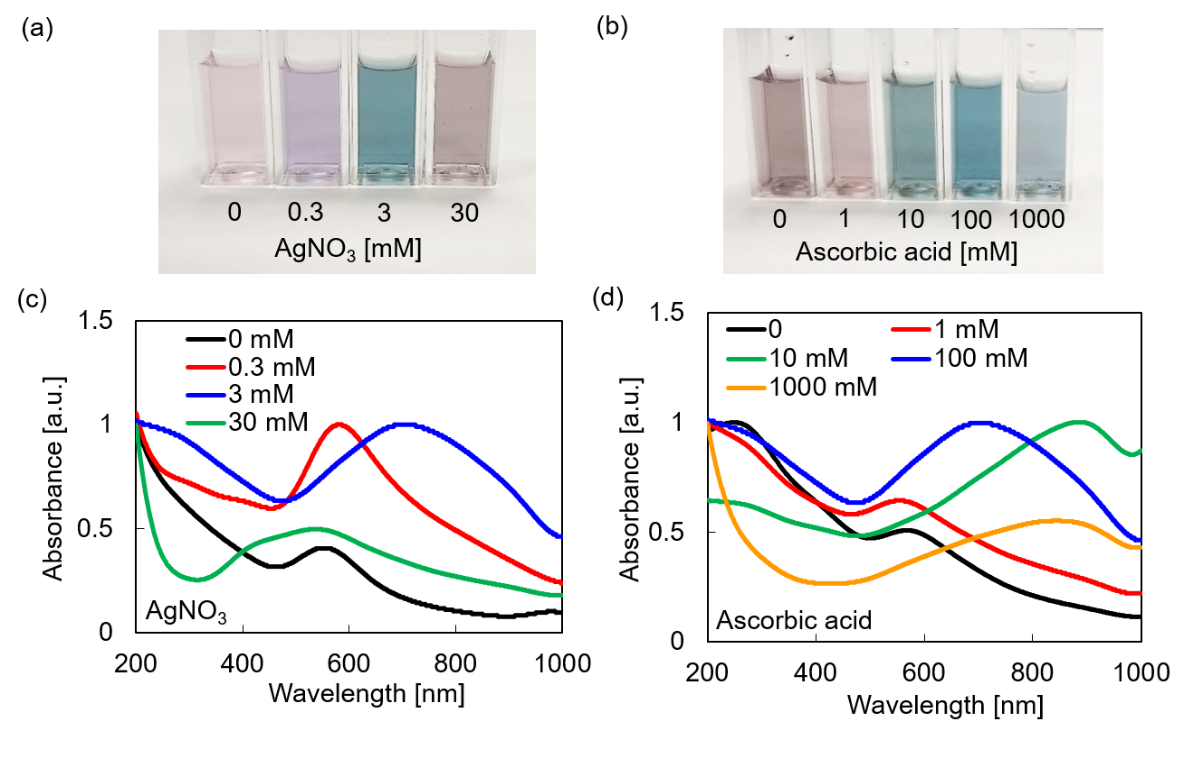


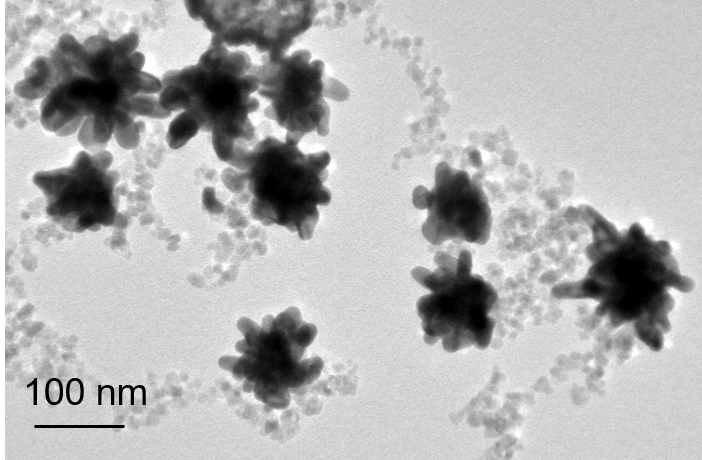


**Figure S2.** A TEM image of MNP@Au nanostars with higher magnification.


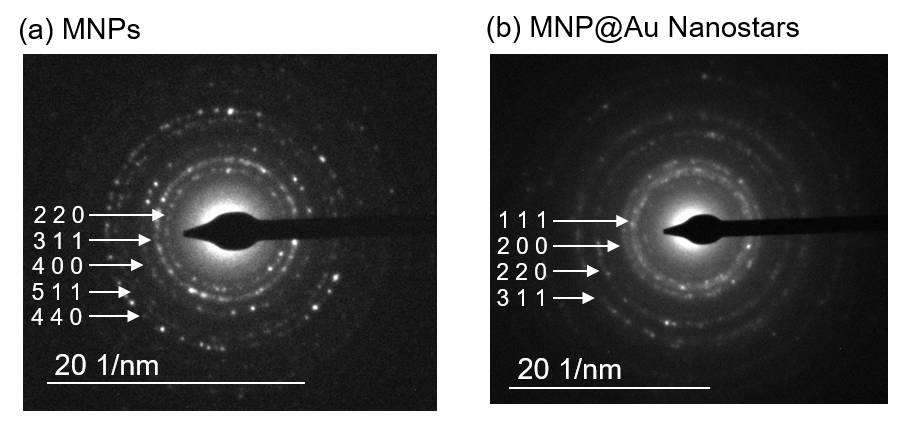


**Figure S3.** Selected area electron diffraction (SAED) patterns of (a) MNPs and (b) MNP@Au nanostars.


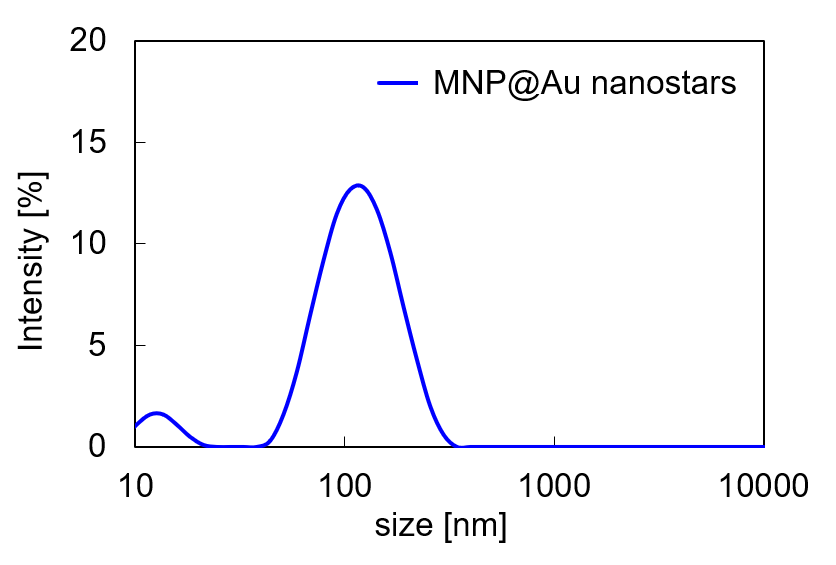


**Figure S4.** Hydrodynamic size distribution of MNP@Au nanostars.


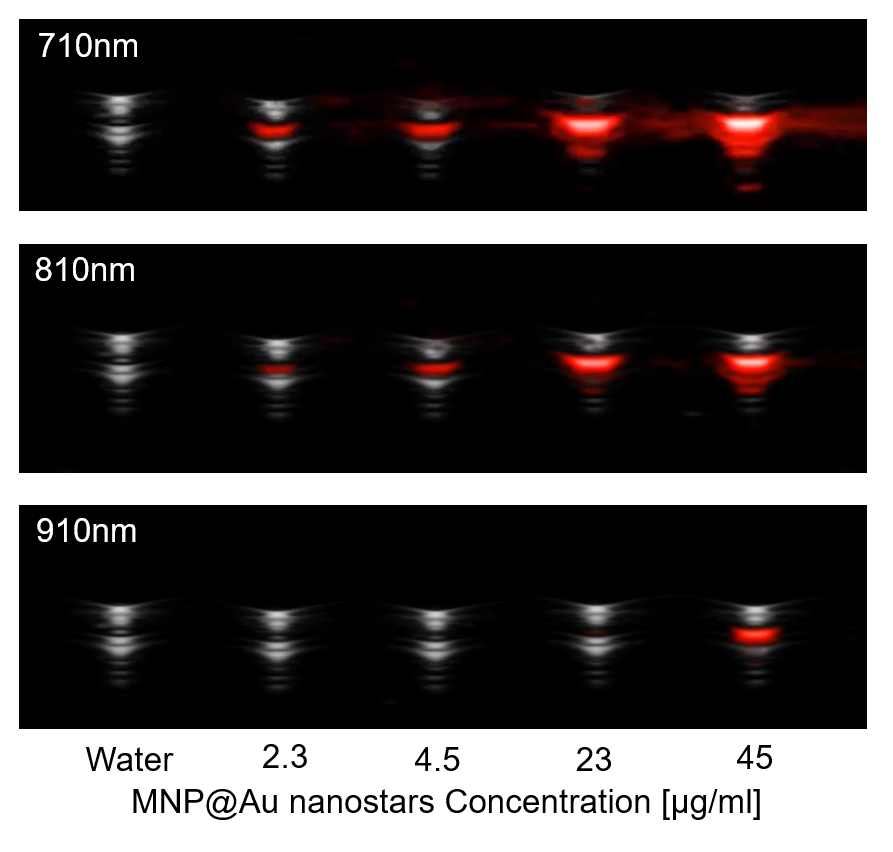


**Figure S5.** PA images of MNP@Au nanostars at different wavelength.


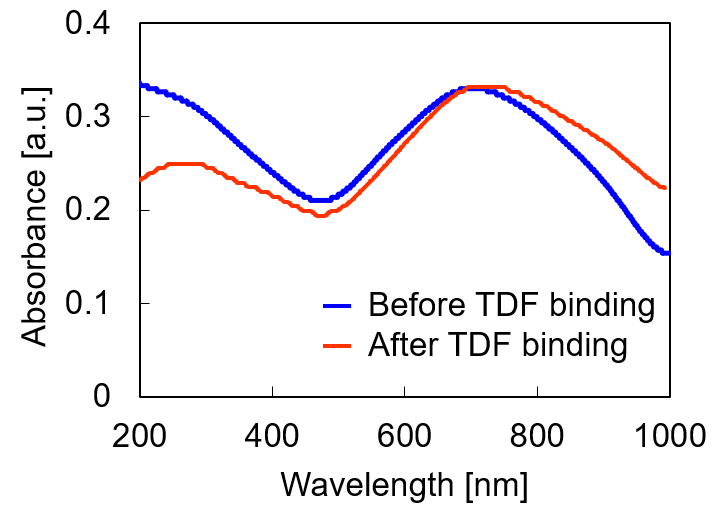


**Figure S6.** Absorbance spectra of MNP@Au nanostars before and after TDF binding.

**Figure S6.** Absorbance spectra of MNP@Au nanostars before and after TDF binding.
